# Characteristics of Prehospital Death in Trauma Victims

**DOI:** 10.3390/jcm10204765

**Published:** 2021-10-18

**Authors:** Jan Gewiess, Christoph Emanuel Albers, Hans-Christoph Pape, Hannes Bangerter, Wolf-Dieter Zech, Marius Johann Baptist Keel, Johannes Dominik Bastian

**Affiliations:** 1 Department of Orthopaedic Surgery and Traumatology, Inselspital, Bern University Hospital, University of Bern, 3010 Bern, Switzerland; christoph.albers@insel.ch (C.E.A.); hannes.bangerter@outlook.com (H.B.); marius.keel@insel.ch (M.J.B.K.); johannes.bastian@insel.ch (J.D.B.); 2 Department of Trauma, University Hospital of Zurich, 8091 Zurich, Switzerland; hans-christoph.pape@usz.ch; 3 Institute of Forensic Medicine Bern, University of Bern, 3012 Bern, Switzerland; Wolf-Dieter.Zech@irm.unibe.ch

**Keywords:** polytrauma, trauma victims, prehospital death, Injury Severity Score (ISS)

## Abstract

Background: Using Injury Severity Score (ISS) data, this study aimed to give an overview of trauma mechanisms, causes of death, injury patterns, and potential survivability in prehospital trauma victims. Methods: Age, gender, trauma mechanism, cause of death, and ISS data were recorded regarding forensic autopsies and whole-body postmortem CT. Characteristics were analyzed for injuries considered potentially survivable at cutoffs of (I) ISS ≤ 75 vs. ISS = 75, (II) ISS ≤ 49 vs. ISS ≥ 50, and (III) ISS < lethal dose 50% (LD50) vs. ISS > LD50 according to Bull’s probit model. Results: In *n* = 130 prehospital trauma victims (45.3 ± 19.5 years), median ISS was 66. Severity of injuries to the head/neck and chest was greater compared to other regions (*p* < 0.001). 52% died from central nervous system (CNS) injury. Increasing injury severity in head/neck region was associated with CNS-injury related death (odds ratio (OR) 2.7, confidence interval (CI) 1.8–4.4). Potentially survivable trauma was identified in (I) 56%, (II) 22%, and (III) 9%. Victims with ISS ≤ 75, ISS ≤ 49, and ISS < LD50 had lower injury severity across most ISS body regions compared to their respective counterparts (*p* < 0.05). Conclusion: In prehospital trauma victims, injury severity is high. Lethal injuries predominate in the head/neck and chest regions and are associated with CNS-related death. The appreciable amount (9–56%) of victims dying at presumably survivable injury severity encourages perpetual efforts for improvement in the rescue of highly traumatized patients.

## 1. Introduction

The term ‘polytrauma’ has been widely established for patients suffering simultaneous injuries to multiple body regions or organ systems, in which the single injury or its combination is potentially life-threatening (Deutsche Gesellschaft für Unfallchirurgie (DGU)). More recent definitions include a priori assumptions of mortality rates >30% factoring the Injury Severity Score (ISS) and physiological parameters such as hypotension, unconsciousness, acidosis, coagulopathy, and age [1]. Prehospital mortality rates have been reported to reach >50% in polytraumatized patients [2,3]. Recent literature elaborating on epidemiology and specific injury patterns related to mortality in trauma victims focused primarily on in-hospital deaths and deaths during transport of trauma patients [4,5,6]. However, this study aimed to identify the most prevalent causes of death and potentially survivable constellations regarding trauma severity in prehospital trauma victims in order to uncover the quality and evolutional potential of trauma rescue.

According to the World Health Organization (WHO), the three leading causes of death from trauma are road-traffic injuries, self-inflicted violence, and homicide (WHO, 2021). A high number of traumatic prehospital deaths results from fatal trauma implying lethal injury with greatest possible severity across multiple body regions [7]. The ISS first described by Baker et al. in 1974 is commonly used to quantify overall trauma severity [8]. An ISS > 15 is usually adopted to characterize major trauma [9]. Patients with an ISS > 24 are considered to be critically injured [10].

Most deaths in trauma victims are reportedly secondary to central nervous system (CNS) injury, uncontrolled bleeding, airway insufficiency, or multiple organ failure (MOF) [11,12]. Establishing the most probable cause of death in dependency of quickly accessible data (trauma mechanism, injury pattern and injury severity) might give direction to possible treatment strategies even before complete Advanced Trauma Life Support (ATLS) assessment is performed.

Using ISS data obtained from autopsy reports and postmortem CT scan data of trauma victims, this study aimed to give an overview of:

- trauma mechanisms,
- causes of death,
- injury patterns,
- influence of regional trauma severity on causes of death,
- trauma mechanisms, causes of death, and injury patterns in potentially survivable injuries.

## 2. Materials and Methods

### 2.1. Patients and Parameters

Between 01/2005 and 12/2013, forensic autopsies and whole body postmortem CT scans prior to autopsy were undertaken for all trauma-related deaths in the canton Bern, Switzerland at the Institute of Forensic Medicine at the University of Bern (*n* = 489). Victims underwent forensic evaluation ordered by local prosecutors due to suspected non-natural cause of death. Within this 9-year period, 130 patients suffered prehospital death. Forensic examinations and postmortem reports were conducted by board certified forensic pathologists. From CT scans and records, an independent investigator assessed each patient’s age, gender, trauma mechanism, cause of death, and injury severity. Victims were included with trauma mechanisms such as motor vehicle accidents (MVA), suicides, workspace accidents, sports accidents, and agriculture accidents. Among those committing suicide, only victims being run over by a train or falling/jumping from height were included. Other suicides, such as hanging, shooting, burning, and stabbing were excluded. Injury severity was assessed using total ISS, Copes’ ISS intervals (proposed for mitigation of heterogeneity with the rationale of the most severe injury/combination included (ISS 16–24, 25–40, 41–49, 50–66, and 75)) [10], and the Abbreviated Injury Score (AIS98) for the respective ISS body region.

Regional injury severity was represented by the AIS98 categories (0 = none, 1 = minor, 2 = moderate, 3 = serious, 4 = severe, 5 = critical, 6 = maximum/currently untreatable). Calculation of the ISS is based on the sum of squares of the most severely injured three of six different body regions (face, head and neck, chest and thoracic spine, abdomen and lumbar spine, pelvis and extremities, external). External injuries included injuries such as abrasions, lacerations, burns, hypothermia, and electrical injury. The ISS ranges from 3–75. In the case of a category 6 trauma to any body region, the ISS is set to 75.

Injury characteristics of trauma victims sustaining potentially survivable injury were compared to those with unsurvivable injury. The respective graduation of lesser traumatization or potentially survivable injury was performed for (I) salvageable submaximal trauma (ISS < 75) according to Chiara et al. [13], (II) an ISS < 49, as proposed by Sampalis et al. [14] and (III) using Bull’s probit model for the derivation of a lethal dose of 50% (LD50) [15]. Using the formula ‘42 − 0.004167 × age^2^’, potential survivability was considered if the patient’s probability of survival is >50%.

### 2.2. Statistical Analysis

Statistical analysis was performed using R (R Foundation for Statistical Computing, Version 4.1.0 (2021), Vienna, Austria). Data description is performed using the median and interquartile range (Q1–Q3) and absolute and relative frequencies, respectively. Frequencies were compared using Chi^2^-tests and Fisher’s exact test in the case of an expected frequency < 5. Normal distribution was tested using the Shapiro–Wilk test. Pairwise comparisons were performed using *t*-tests for parametric and Mann-Whitney-*U*-tests for non-parametric continuous data. Multivariable analyses were performed using logistic regression. Results are reported as Odds ratios (OR) and corresponding 95% confidence intervals.

## 3. Results

### 3.1. Overview of Trauma Mechanisms and Causes of Death

Within a 9-year period, *n* = 130 trauma victims died before arriving at the emergency department (mean age 45.3 ± 19.5 years; range 16–92). Seventy four percent victims were of male gender. The most common trauma mechanism was MVA (54%), followed by suicide (18%), industrial/workspace accidents (9%), sports accidents (7%), and agriculture accidents (2%). Among sports accidents, paragliding, parachuting, and base-jumping were most common followed by skiing/snowboarding or hiking and sledding or climbing. The most frequently coded cause of death was CNS injury (52%), followed by uncontrolled bleeding (42%), airway compromise (32%) and MOF (29%). A single cause of death was found in *n* = 78 (60%), a combination of 2 in *n* = 37 (29%), a combination of 3 in *n* = 12 (9%), and a combination of four in *n* = 3 (2%) patients. Numerical summaries of demographic data, trauma mechanisms, and causes of death are given in Table 1.

Median ISS was 66 in 130 deceased. According to causes of death, median total ISS was highest in (combined) CNS injuries (65) and lowest in (combined) MOF (56). Compared to victims of CNS injuries, total ISS was significantly lower in those dying of airway compromise (*p* = 0.019) or those dying of MOF (*p* = 0.036). According to trauma mechanism, median ISS was highest in suicides (66) and lowest in sports accidents (50). Differences were not significant (*p* > 0.16). Descriptive statistics of ISS distributions are shown in Table 2.

### 3.2. Overview of Injury Patterns

**Figure 1:**
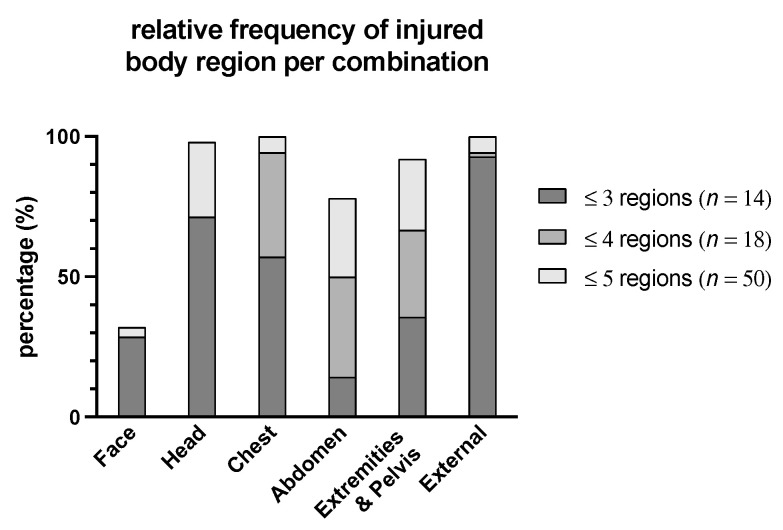
Relative distribution of injured body regions according to ISS per injury combination in prehospital trauma-related deaths.

No patient had injuries to only one body region. Two body regions were injured in *n* = 3 (2%), three in *n* = 14 (11%), four in *n* = 18 (14%), five in *n* = 50 (39%), and six in *n* = 45 (35%) patients. Face trauma was present in *n* = 70 (54%), head and neck trauma in *n* = 117 (90%), thoracic trauma in *n* = 122 (94%), abdominal trauma in *n* = 96 (74%), pelvic and extremity trauma in *n* = 108 (83%), and external injury in *n* = 127 (98%). Depending on the number of injury combinations, different body regions were predominantly injured (Figure 1). For example, abdominal injuries were rather infrequent when there were ≤3 different body regions affected (14%) compared to when ≤5 regions were affected (78%). The frequency of face or external injury was comparable, regardless of the number of injured body regions.

### 3.3. Overview of Injury Severity

According to Copes’ ISS intervals, *n* = 6 (5%) presented with an ISS of 1–24, *n* = 22 (17%) with an ISS of 24–49, *n* = 45 (35%) with an ISS of 50–74, and *n* = 57 (44%) with an ISS of 75. According to the ISS body regions, severity was critical (median = 5, Q1 = 3.25, Q3 = 6) in head/neck injuries, minor (median = 1, Q1 = 0, Q3 = 3) in face injuries, critical (median = 5, Q1 = 4, Q3 = 5) in chest injuries, severe (median = 4, Q1 = 0, Q3 = 5) in abdomen injuries, serious (median = 3, Q1 = 2, Q3 = 4) in pelvis/extremity injuries, and moderate (median = 2, Q1 = 2, Q3 = 2) in external injuries.

**Figure 2:**
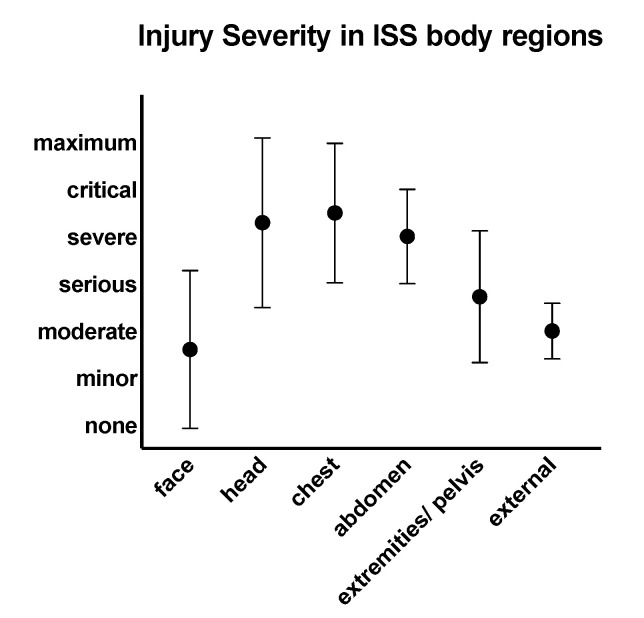
Median and 95% CI of injury severity across ISS body regions. Symbol: median, whiskers: 95% CI.

Face injuries were significantly less severe compared to injuries across all other body regions except for external (*p* < 0.001, Figure 2). Median head and neck injury and chest injury both were significantly more severe than injuries to abdomen, extremities, and external (*p* < 0.001). Abdominal injuries were significantly more severe compared to external injuries (*p* < 0.001). Extremity injuries were significantly more severe compared to external injuries (*p* < 0.002). Descriptive statistics of injury severity in respective ISS body regions are provided in Table 2 and Figure 2.

### 3.4. Influence of Regional Trauma Severity on Causes of Death

**Figure 3:**
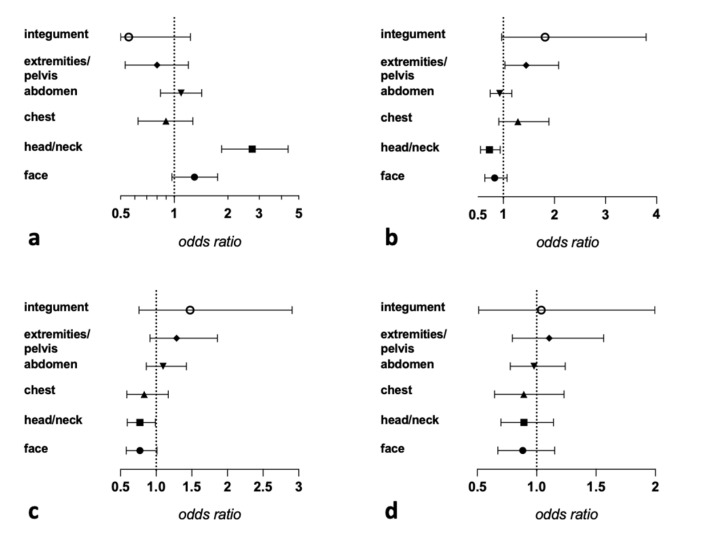
Coefplots (Odds ratios with 95% Wald confidence limits) for (**a**) CNS injury, (**b**) exsanguination, (**c**) airway compromise, (**d**) MOF. Most CI indicate no clear monodirectional relation between an increasing regional injury severity and a specific reason of death.

Relative risk (OR) of succumbing a specific cause of death relative to an increasing trauma severity to the respective ISS body region is shown in Figure 3. Among these, the clearest indicator for an increased OR for a specific cause of death is an increased trauma severity in the head/neck region for the CNS-injury related death (OR 2.7, CI 1.8–4.4). For the remaining causes of death, a clear monodirectional relation to regional injury severity could not be established.

### 3.5. Comparison of Trauma Victims with Potentially Survivable Injuries versus Trauma Victims with Non-Survivable Injuries

**Figure 4:**
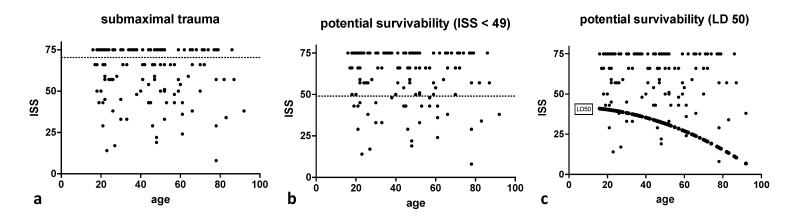
Age versus Injury Severity Score (ISS) for trauma victims with submaximal trauma/ISS < 75 (**a**), potentially survivable injuries/ISS < 49 (**b**), and age versus Injury Severity Score (ISS) and LD50 derived from Bull’s probit analysis (**c**). Submaximal trauma was present in 56% and potentially survivable trauma in 22% and 9%, respectively.

*N* = 73 (56.2%) victims died with an ISS < 75 and *n* = 57 (43.8%) died with an ISS of 75. *N* = 28 (22%) victims sustained potentially survivable trauma characterized by an ISS < 49. According to Bull’s model, *n* = 11 (9%) patients sustained potentially survivable trauma (Figure 4). Median ISS was 54 in the case of submaximal trauma, 37 for victims with an ISS < 49, and 24 for victims with an ISS < LD50. Age and gender of victims were comparable among respective groups for all three approaches (*p* > 0.4). Regarding trauma mechanisms, there were no significant differences between victims of maximal/submaximal or unsurvivable/potentially survivable trauma (Table 3). However, people committing suicide more frequently sustained maximal injuries compared to other trauma mechanisms (*p* < 0.07). People dying in sports accidents rather sustained submaximal (*p* < 0.08) or potentially survivable injuries (*p* < 0.17).

**Figure 5:**
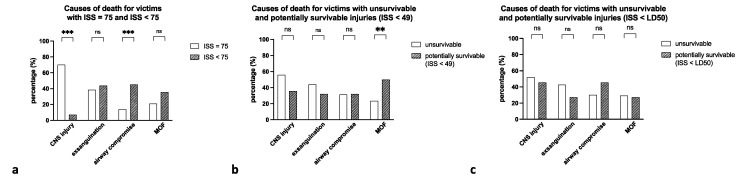
Relative frequencies of causes of death for (**a**) maximal (clear) vs. submaximal (patterned) trauma, (**b**) unsurvivable (ISS > 49, clear) vs. potentially survivable (ISS < 49, patterned) trauma, and (**c**) unsurvivable (ISS > LD50, clear) vs. potentially survivable (ISS < LD50, patterned) trauma. Relative frequency of CNS injury was always higher in the suspected higher traumatized group but reached significance only in the comparison of maximal to submaximal trauma (**a**). In turn, airway compromise was more frequently observed in victims sustaining potentially survivable injury with significance only when comparing maximal to submaximal trauma (**a**). Similarly, MOF was more frequent in submaximal (**a**) and potentially survivable (**b**,**c**) trauma. *** *p* < 0.001, ** *p* < 0.01, ns: not significant.

Relative frequencies of causes of death varied when comparing maximal to submaximal and unsurvivable to potentially survivable trauma. In the case of the respective higher suspected traumatization (maximal and unsurvivable injury), CNS injury was the most common cause of death (>52%), followed by exsanguination (>40%). For the suspected lesser traumatization (submaximal and potentially survivable injuries), frequencies of airway compromise and MOF were generally increased (up to 80%). A comparison of causes of death for the three approaches is presented in Figure 5.

**Figure 6:**
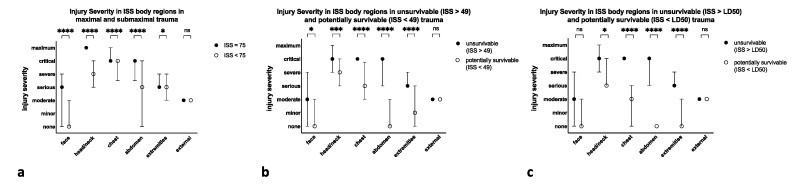
Median and IQR of injury severities according to the AIS-98 within the respective ISS body regions for prehospital deaths and submaximal (ISS < 75) injury (**a**), unsurvivable (ISS > 49) and potentially survivable (ISS < 49) injury (**b**), and unsurvivable (ISS > LD50) and potentially survivable (ISS < LD50) injury (**c**). There were significant differences in all body regions except for the external. **** *p* < 0.0001, *** *p* < 0.001, * *p* < 0.5, ns not significant. Symbol: median, whiskers: IQR.

Overall, injury severity of specific body regions was lower in the suspected lesser traumatization (submaximal and potentially survivable injuries. Table 4 and Figure 6). There were significant differences for all body regions, except for the external. Especially, injury severity to the chest and abdomen was significantly lower in submaximal and potentially survivable injuries (*p* < 0.001).

## 4. Discussion

Means to identify injury patterns and causes of death in deceased patients are autopsy and postmortem CT imaging [16,17]. From a forensic perspective, autopsy remains the gold standard for the evaluation of injury patterns. Postmortem CT imaging can be a useful addition, especially for the evaluation of osseous injuries to the facial bones, spine, and extremities [18]. Thus, in Switzerland, postmortem CT scans are routinely obtained in these cases. Using ISS data obtained from congruously unique postmortem whole-body CT data and autopsies of prehospital trauma victims within a nine-year period, we were able to evaluate injury patterns, trauma mechanisms and corresponding causes of death and to identify potentially survivable relations. Only few studies evaluated prehospital death in trauma victims in the past decades [19,20,21,22,23,24,25].

Potentially treatable injuries in the prehospital phase included all injuries categorized by an AIS ≤ 5. Exemplary, for the head/neck region, these range from cerebral concussion (AIS = 1), via skull fracture (AIS = 2), traumatic aneurysm (AIS = 3), artery occlusion (AIS = 4), to brainstem contusion (AIS = 5). In contrast, prehospital medical treatment was not carried out in trauma victims presenting with the appearance of certain signs of death such as livor mortis or injuries not compatible with life. While the former may rather be expectable in intentional infliction (suicides, homicides), the latter are implied by the greatest possible injury as categorized by an AIS = 6 (such as decapitation, massive scull destruction, total severance of the aorta, avulsed liver, or torso transection).

We confirmed earlier findings of predominantly male, middle-aged patients dying from trauma in the prehospital environment [19,20,21,23,25], with the predominant trauma mechanism being MVA [21,23,25,26]. In our cohort, suicide was the second-most common trauma mechanism. As committed suicide by trauma is intended to result in death (e.g., maximal trauma, avoidance of public detection), appropriate rationale for (non-) timely initiation of (in-) adequate therapy might be negligible. Likewise, it demonstrates the comparative potential in the rescue of mechanistic scenarios not implying similar preconditions.

Recently, the acceptance of a potentially historical trimodal temporal distribution of trauma mortality has been questioned and might have to be corrected towards a rather unimodal distribution relative to posttraumatic days [6]. In a cohort of 277 victims of road traffic injuries, Pfeifer et al. reported on a mortality rate of 78% within the first six hours. Furthermore, they were able to show an association between different injury patterns and temporal mortality distribution.

Our findings of a high percentage of lethal sports accidents have not yet been reported. These are especially worrying given the relatively lower ISS and younger age of these patients. The lower ISS in sports-related deaths essentially arising from exsanguination and MOF implicates preventability (inadequate primary treatment or long transportation times) and contrasts with earlier findings, where MOF related deaths were reported to be attributable to the in-hospital mortality of predominantly older trauma victims [12]. Differences in pre-clinical mortality might be explicable in view of geographical diversity [6]. Exemplarily, the reported median time of 2–3 h to death from hemorrhagic shock substantiates the dependability of surviving on geographics and transport times [27,28]. However, given the predominance of mountain sports accidents in our cohort, shortening search and rescue times might confront with the security of the emergency personnel.

Similar to our findings, earlier reports on injury patterns in prehospital trauma deaths showed a prevalence of >50 to >80% for head and neck and chest injuries [19,20,25,26]. Severity characterization of injuries to different body regions has hardly been performed. Ryan et al. [20] reported the most severe injury was most commonly located either in the head or chest region. Falconer [26] reported the head/neck region to be the single largest number of AIS 6 scores, which is reflected by the comparatively highest AIS scores in this region in our study. For each increasing AIS severity category of head and neck injury, we found the odds of dying from CNS injury to be multiplied by 2.7. Over the past 30 years, CNS injury continues to be the main cause of death in prehospitally deceased [12] as well as in polytraumas admitted to the hospital [4,6,29]. Except for the head and neck trauma severity and lethality of CNS injury, our multivariate analysis did not productively result in clear relations of injury severities to selected body regions and the prediction of a specific cause of death. This might be secondary to the coexistence of multiple causes of death or the great severity of injuries to multiple body regions.

To date, ISS values of prehospitally deceased trauma patients have only been reported in few studies [20,23]. While Hussain et al. found the majority of trauma related prehospital deaths in the ISS range of 21–50 (61.2%), Ryan et al. showed the main part to have an ISS of 75 (37%). This is in line with our results (43.9% having an ISS of 75).

The relative number of patients dying in the prehospital setting with the maximum ISS of 75 seems to increase in the past decades. In 1994, Hussain et al. found 19.1% of their study cohort to have the maximum ISS of 75 [23]. Later, Papadopoulos et al. found an AIS-6 injury (ISS = 75 by definition) in 35% of patients in their cohort [22]. Ryan et al. investigated injury patterns and preventability in prehospital motor vehicle crash fatalities and found 37% of the study population to have the maximum ISS of 75 [20]. In this study, there were 44% of prehospital deaths having an ISS of 75.

This might have two reasons: On the one hand, early treatment and transport time might have improved and thus the relative number of patients surviving the prehospital phase increases compared to those with lethal injury. This would be in line with the low number of patients with an ISS < 16 in our cohort (1.5%). On the other hand, and in light of the high incidence of lethal MVA, the energy of trauma mechanisms in trauma victims might have increased.

The performance of the ISS to quantify injury severity and potential survivability remains questionable, especially, when considering the representation of multiple injuries to the same body region [1]. In line with the recent polytrauma definition, an ISS-only based approach alone cannot specify the really critically ill trauma patients [30]. Sampalis et al. [14] proposed ISS groupings to classify survivability (survivable ISS < 24; potentially survivable ISS 25–49; non-survivable ISS > 49). Age has been repeatedly shown to influence ISS based mortality prediction [15,31] and was therefore integrated in the LD50 consideration according to Bull [15,30]. Especially in patients with severe injuries, ISS might not be a valid instrument to predict mortality [32].

Defining preventable death from limited patient data remains a challenge and proposed rates of preventability have to be interpreted carefully given their methodological formation. Depending on the resorted threshold being submaximal injury, ISS < 49 or ISS < LD50, or an expert panel, potential survivability rates in trauma victims reportedly range from 15–47% (9–56% in this study) [19,20,21,22,23,24]. Our results demonstrate some clear trends regardless of the threshold: While non-survivability was determined essentially by CNS injury, trauma victims sustaining potentially survivable injuries primarily died from airway compromise and MOF. As expected, and in line with our multivariate analysis, injuries to the head and neck region were significantly more severe in maximal and unsurvivable injuries.

To our knowledge, this study is the first to use postmortem ISS obtained from autopsies combined with whole-body CT scans of an adequate sample size for analysis of prehospital death in trauma victims. Another strength is the long period of the study. Thus, we were able to provide a detailed breakdown of the injuries that led to death.

Nevertheless, the heterogenous character of injury patterns and the coexistence of several causes of death in 40% of cases limits their attributability. A further limitation of the data acquisition is that no information about any prehospital management is available. However, given the high rate of definitively unsurvivable trauma (44% of cases presenting with an ISS = 75) combined with the aforementioned heterogeneity of injury patterns, it remains questionable whether further analysis of individual prehospital treatment might gain any insights for preclinical rescue personnel.

## 5. Conclusions

Traumatic prehospital death is most commonly secondary to MVA. In these patients, (lethal) injuries predominate in the head/neck and chest region. CNS injury is the most common cause of death and is associated with the severity of injuries to the head and neck.

All estimation of preventable death rate from limited data must be a rough approximation. Whatever value is adopted, it is certain there is still potential for improvement in the rescue of polytraumas as there is an appreciable amount of victims dying from stoppable or reversible causes at presumably survivable overall injury severity.

## Figures and Tables

**Table 1:** Frequencies and numerical summaries of demographic data, trauma mechanisms, and causes of death.

	***n***	Mean Age (SD, IQR)	*n* f/m (%)	Median ISS (Q1, Q3)	*n* CNS Injury (%)	*n* Exsanguination (%)	*n* Airway Compromise (%)	*n* MOF (%)
Overall	130	45.3 (19.49, 32.75)	34/96 (26.15/73.85)	66 (50, 75)	67 (51.54)	54 (41.54)	41 (31.54)	38 (29.23)
MVA	70	48.48 (20.4, 32.00)	16/54 (22.9/77.1)	66 (54.75, 75)	36 (51.4)	32 (45.7)	20 (28.6)	22 (31.4)
Suicide	23	40.48 (17.06, 23.5)	11/12 (47.8/52.2)	75 (62.5, 75)	12 (52.2)	10 (43.5)	10 (43.5)	6 (26.1)
Industrial/ workspace	12	42.25 (14.06, 22.25)	0/12 (0/100)	62.5 (47.75, 75)	10 (83.3)	4 (33.3)	4 (33.3)	4 (33.3)
Sports	9	36.11 (15.99, 27)	3/6 (33.3/66.7)	50 (43, 66)	1 (11.1)	4 (44.4)	2 (22.2)	5 (55.6)
Agriculture	3	64 (17.78, 17)	1/2 (33.3/66.7)	57 (53.5, 66)	1 (33.3)	1 (33.3)	1 (33.3)	1 (33.3)

SD, Standard deviation; IQR, interquartile range; f/m, female to male ratio; ISS, Injury Severity Score; CNS, central nervous system; MOF, multiple organ failure; MVA, motor vehicle accident.

**Table 2:** Median (Q1, Q3) total ISS and severity in respective ISS body regions according to trauma mechanisms and causes of death.

	*n* (%)	Median Total ISS (Q1, Q3)	Median ISS Head/Neck (Q1, Q3)	Median ISS face (Q1, Q3)	Median ISS Chest (Q1, Q3)	Median ISS Abdomen (Q1, Q3)	Median ISS Pelvis/Extremities (Q1, Q3)	Median ISS External (Q1, Q3)
Overall	130	66 (50, 75)	5 (3.25, 6)	1 (0, 3)	5 (4, 5)	4 (0, 5)	3 (2, 4)	2 (2, 2)
MVA	70 (54)	66 (54.75, 75)	4 (4, 6)	2 (0, 3.75)	5 (4, 6)	4 (0, 5)	3 (2, 4)	2 (2, 2)
Suicide	23 (18)	75 (62.5, 75)	6 (3.5, 6)	2 (0, 4)	5 (4, 5)	5 (3, 5)	3 (3, 4)	2 (2, 2)
Workspace	12 (9)	62.5 (47.75, 75)	5 (3, 6)	0 (0, 2.25)	5 (3.75, 5)	5 (4.25, 5)	3 (0, 4)	2 (2, 2)
Sports	9 (7)	50 (43, 66)	4 (3, 5)	1 (0, 3)	5 (4, 5)	0 (0, 5)	3 (2, 3)	2 (2, 2)
Agriculture	3 (2)	57 (53.5, 66)	3 (1.5, 4.5)	0 (0, 0.5)	5 (5, 5)	4 (3, 4.5)	4 (3.5, 4)	2 (2, 2)
CNS injury	67 (52)	75 (57, 75)	6 (5, 6)	2 (0, 4)	5 (4, 5)	5 (1, 5)	3 (2, 4)	2 (2, 2)
Exsanguination	54 (42)	66 (57, 75)	4 (3, 5)	0 (0, 2.75)	5 (5, 6)	4 (3, 5)	3 (3, 4)	2 (2, 2)
Airway compromise	41 (32)	59 (50, 66)	4 (3, 5)	0 (0, 2)	5 (5, 5)	4 (3, 5)	3 (2, 4)	2 (2, 2)
MOF	38 (29)	61.5 (43, 75)	4 (3, 5)	0 (0, 2)	5 (4, 5)	3 (0.5, 5)	3 (2, 4)	2 (2, 2)

Q1, First quartile; Q3, third quartile; ISS, Injury Severity Score; CNS, central nervous system; MOF, multiple organ failure; MVA, motor vehicle accident.

**Table 3:** Absolute (relative) frequencies and comparison of demographic data, trauma mechanisms, and causes of death for trauma victims with submaximal trauma/ISS < 75, potentially survivable injuries/ISS < 49, and potentially survivable (ISS < LD50 as derived from Bull’s probit analysis). *N* (ISS < 75) = 73, *n* (ISS < 49) = 28, *n* (ISS < LD50) = 11.

	**Overall (%)**	**ISS < 75**	**ISS 75**	***p*-Value**	**ISS < 49**	**ISS > 49**	***p*-Value**	**ISS < LD50**	**ISS > LD50**	***p*-Value**
Median ISS	66	54	75		37	75		24	66	
Mean age	46	47	44	0.664	47	45.5	0.626	33	47	0.403
*n* female/male (%)	34/96 (26.15/73.85)	21/52 (28.8/71.2)	13/44 (22.8/77.2)	0.443	9/19 (32.1/67.9)	25/77 (24.5/76)	0.568	4/7 (36.4/63.6)	30/89 (25.2/75)	0.477
*n* MVA (%)	70 (54)	38 (52.1)	32 (56.1)	0.643	13 (46.4)	57 (55.9)	0.374	4 (36.4)	66 (55.5)	0.224
*n* suicide (%)	23 (18)	9 (12.3)	14 (24.6)	0.07	3 (10.7)	20 (19.6)	0.403	1 (9.1)	22 (18.5)	0.435
*n* workspace (%)	12 (9)	8 (11)	4 (7)	0.441	3 (10.7)	9 (8.8)	0.721	1 (9.1)	11 (9.2)	1
*n* sports (%)	9 (7)	8 (11)	1 (1.8)	0.077	4 (14.3)	5 (4.9)	0.1	2 (18.2)	7 (5.9)	0.169
*n* agriculture (%)	3 (2)	2 (2.7)	1 (1.8)	1	0 (0)	3 (2.9)	1	0	3 (2.5)	1
*n* CNS injury (%)	67 (52)	27 (37)	40 (70.2)	<0.001	10 (35.7)	57 (55.9)	0.059	5 (45.5)	62 (52.1)	0.673
*n* exsanguination (%)	54 (42)	32 (43.8)	22 (38.6)	0.548	9 (32.1)	45 (44.1)	0.255	3 (27.3)	51 (42.9)	0.36
*n* airway compromise (%)	41 (32)	33 (45.2)	8 (14)	<0.001	9 (32.1)	32 (31.4)	0.938	5 (45.5)	36 (30.3)	0.321
*n* MOF (%)	38 (29)	26 (35.6)	12 (21.1)	0.07	14 (50)	24 (23.5)	0.007	3 (27.3)	35 (29.4)	1

**Table 4:** Means (SD, IQR) of total ISS and of injury severity to the respective body region.

	Overall	ISS < 75	ISS 75	*p*-Value	ISS < 49	ISS > 49	*p*-Value	ISS < LD50	ISS > LD50	*p*-Value
Face	1 (0, 3)	0 (0, 2)	3 (0, 4)	<0.001	0 (0, 2)	2 (0, 4)	0.03	0 (0, 1.5)	2 (0, 4)	0.148
Head/neck	5 (3.25, 6)	4 (3, 5)	6 (6, 6)	<0.001	4 (3, 5)	5 (4, 6)	<0.001	3 (3, 5)	5 (4, 6)	0.024
Chest	5 (4, 5)	5 (4, 5)	5 (5, 6)	<0.001	3 (2, 4.5)	5 (5, 5)	<0.001	2 (0, 2.5)	5 (5, 5)	<0.001
Abdomen	4 (0, 5)	3 (0, 5)	5 (4, 5)	<0.001	0 (0, 2)	5 (3, 5)	<0.001	0 (0, 0)	5 (3, 5)	<0.001
Pelvis and extremities	3 (2, 4)	3 (2, 4)	3 (3, 4)	0.022	1 (0, 3)	3 (3, 4)	<0.001	0 (0, 2)	3 (3, 4)	<0.001
External	2 (2, 2)	2 (2, 2)	2 (2, 2)	0.155	2 (2, 2)	2 (2, 2)	0.962	2 (2, 2)	2 (2, 2)	0.122

## Data Availability

The data presented in this study are available on request from the corresponding author. The data are not publicly available due to privacy reasons.
